# Characterisation of a Novel White Laccase from the Deuteromycete Fungus *Myrothecium verrucaria* NF-05 and Its Decolourisation of Dyes

**DOI:** 10.1371/journal.pone.0038817

**Published:** 2012-06-08

**Authors:** Dan Zhao, Xi Zhang, Daizong Cui, Min Zhao

**Affiliations:** 1 Department of Microbiology, Life Science College, Northeast Forestry University, Harbin, China; 2 Laboratory of Microbiology, College of Life Science, Heilongjiang University, Harbin, China; Centre National de la Recherche Scientifique, France

## Abstract

A novel ‘white’ laccase was purified from the deuteromycete fungus, *Myrothecium verrucaria* NF-05, which was a high laccase-producing strain (40.2 U·ml^−1^ on the thirteenth day during fermentation). SDS-PAGE and native-PAGE revealed a single band with laccase activity corresponding to a molecular weight of approximately 66 kDa. The enzyme had three copper and one iron atoms per protein molecule determined by ICP-AES. Furthermore, both UV/visible and EPR spectroscopy remained silence, indicating the enzyme a novel laccase with new metal compositions of active centre and spectral properties. The N-terminal amino acid sequence of the purified protein was APQISPQYPM. Together with MALDI-TOF analysis, the protein revealed a high homology of the protein with that from reported *M. verrucaria*. The highest activity was detected at pH 4.0 and at 30°C. The enzyme activity was significantly enhanced by Na^+^, Mn^2+^, Cu^2+^ and Zn^2+^ while inhibited by DTT, NaN_3_ and halogen anions. The kinetic constant (*K*m) showed the enzyme was more affinitive to ABTS than other tested aromatic substrates. Twelve structurally different dyes could be effectively decolourised by the laccase within 10 min. The high production of the strain and novel properties of the laccase suggested its potential for biotechnological applications.

## Introduction

Laccase (EC 1.10.3.2, benzenediol: oxygen oxidoreductase) is a multicopper oxidase that has become an important industrially relevant enzymes that is used in paper-pulp bleaching [1], synthetic dyes decolourisation [2], bioremediation [3], chemical analysis and bioelectronics [4]. Laccases are widely distributed among in plants [5], bacteria [6] and especially fungi [7]. In fungi, laccases are produced by many ascomycetes [2], [8], basidiomycetes [9] and some deuteromycetes [10], [11], [12] which are involved in plant pathogenesis, pigmentation, detoxification and lignin degradation [7]. The deuteromycete fungus *Myrothecium verrucaria* is widely industrially used to produce bilirubin oxidase. The only reported laccase from this species was defined as an alkaliphilic laccase without a detailed purification and characterization [13].

Normal copper-containing laccases contain three types of copper that can be distinguished using UV/visible and EPR spectra. T1 copper gives a blue color to the protein from an absorbance at about 600 nm and is EPR detectable. T2 copper confers no color, but is EPR detectable. T3 copper is a pair of copper atoms that give a weak absorbance in the near UV and have no EPR signal [14]. However, laccases with a differently structured active site are also described in literatures [15], [16]. Enzymes lacking the maximum around 600 nm in the absorption spectrum are usually classified as ‘yellow’ or ‘white’ laccases because they have the catalytic activity inherent in typical ‘blue’ laccases [17]. Different active center might confer these laccases different properties of interest.

In the previous work, we isolated the deuteromycete, *M.verrucaria* NF-05, from the soil of a pine forest in the Liangshui Nature Reserve (47°10′N, 128°53′E) in China in November 2009 [18]. The present study described the purification and characterization of a novel ‘white’ laccase from the strain. The metal content, UV/visible and EPR spectra characteristics, N-terminal sequence and MALDI-TOF analysis were elaborated. The effects of pH, temperature, metal ions, putative inhibitors, organic solvents and reaction with different aromatic compounds on the purified laccase were investigated. In addition, the applications of the purified laccase in the decoloursation of various dyes were discussed.

## Results and Discussion

### Production and purification of extracellular laccase

**Figure 1:**
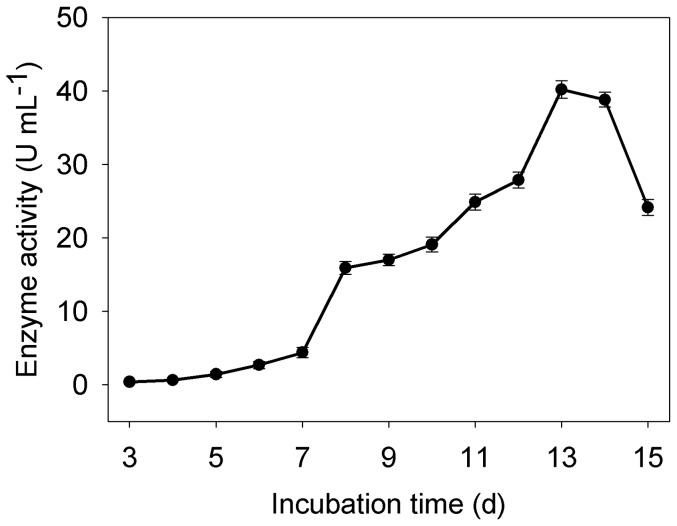
Production of extracellular laccase by *M.verrucaria* NF-05. Results represents means of three experiments, and error bars indicates ± standard error.

**Figure 2:**
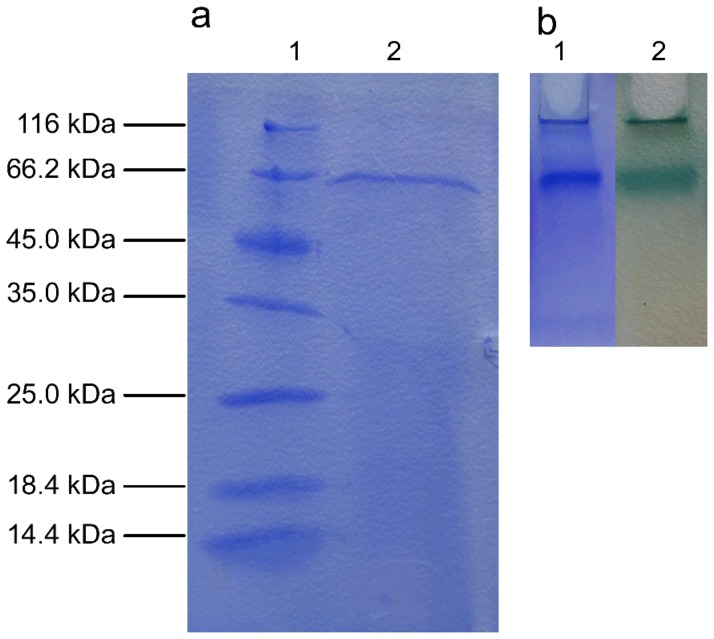
SDS–PAGE (a) and native PAGE (b) of purified laccase from *M.verrucaria* NF-05. (a) Lane 1: denatured protein marker, Lane 2: purified laccase; (b) Lane 1: purified laccase with Coomassie Brilliant Blue R-250staining, Lane 2: purified laccase with ABTS staining.

The production of laccase by *M.verrucaria* NF-05 was performed in shaking flask cultures at 140 rpm, 30°C that were induced with 1 mM copper for 15 days. The amount of laccase production increased rapidly after 7 days and the maximum activity was recorded on day 13 (40.2 U·ml^−1^) (Fig. 1). The laccase activity dropped sharply at day 15. No activity of bilirubin oxidase was detected in the fermentation liquid. The property of laccase to maintain a high production over a short time is interesting from the industrial point of view. The purification achieved a 34.7-fold increase in the activity with a yield of 15.7% (Table 1). The SDS-PAGE revealed the purity of the sample and a molecular weight of 66 kDa (Fig. 2a). The green band on native-PAGE was oxidized ABTS which indicated the laccase activity (Fig. 2b). Both SDS-PAGE and native PAGE suggested that this enzyme is a monomeric protein (Fig. 2). The only reported purified laccase from *M. verrucaria* 24G-4 is 62 kDa [13].

**Table 1:** Steps in protein purifying to homogeneity of *M.verrucaria* NF-05 cultures.

Purification step	Volume (ml)	Activity (U ml^−1^)	Total activity (U)	Total protein (mg)	Specific activity (U mg^−1^)	Yield (%)	Fold purification
Crude culture	200.0	38.6	7720.0	170.8	45.2	100.0	1.0
(NH_4_)_2_SO_4_ precipitation	20.0	232.6	4651.3	34.3	135.6	60.3	3.0
DEAE-Cellulose column	10.0	320.9	3208.6	3.9	821.0	41.6	18.2
Sephadex G-75 column	10.0	121.1	1211.1	0.8	1568.8	15.7	34.7

### Spectral properties of the purified protein

**Figure 3:**
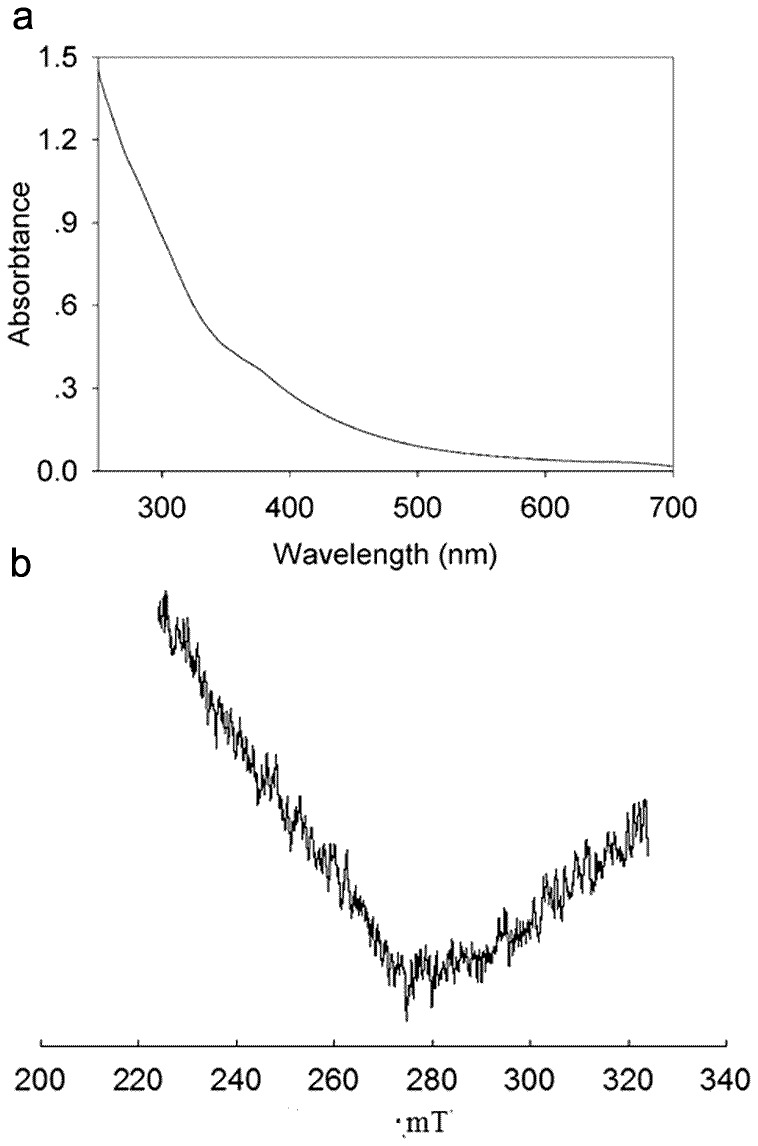
UV–visible absorption (a) and electron paramagnetic resonance spectra (b) of laccase from *M.verrucaria* NF-05 in 10 mM citric acid buffer (pH 4.0)

The inductively coupled plasma atom emission spectrometry (ICP-AES) showed that the enzyme contained copper and irons ions with a ratio of 3∶1. The quantitative analysis resulted in 3.08±0.3 copper atoms and 0.95±0.2 iron atoms per protein molecule. The UV-visible spectrum of purified enzyme gave an atypical spectrum (Fig. 3a). There is no peak around 600 nm corresponding to a T1 blue copper which was consonant with the colorless solution of the purified enzyme. Simultaneously, there is no shoulder at 330 nm corresponding to a T3 binuclear copper. The EPR spectra also remained silence thus no T1 and T2 Cu signal were detectable (Fig. 3b). As a rule, the Cu^2+^ renders the blue color of a liquid. The Cu^2+^ whose electron configuration is d^9^ engenders a d-d transition and absorbs visible light under the act of ligands so as to appear color. Excluding the contamination during purification, the colorlessness of the protein in this research could result from the change of valence state of Cu^2+^. In addition, no EPR signal was detected owing to Fe^2+^ whose electron configuration presented a low-spin state.

Though it was not clear the arrangement of these metal atoms and which copper was replaced by iron, it could be deduced that the lack of absorption in visible light range probably resulted from the existence of incomplete oxidation state of copper (Cu^+^), which had a fully occupied electron configuration of d^10^ and no d-d transition could take place. The easiness of electron transfer in active center of the laccase which was conferred by the incomplete oxidation of metal ions might render the protein extra high activity. The partial silence of spectrum and EPR detection have been found in the ‘white’ laccase POXA1 from *Pleurotus ostreatus* which contained only one copper atom, together with two zinc and one iron atoms per molecule [15], ‘white’ laccase from *Phellinus ribis* which contained one copper, one manganese and two zinc atoms [19]. To sum up, the metal content in active center and total silence on UV/visible and EPR spectra indicated the purified ‘white’ laccase was different from all reported laccases.

The N-terminal amino acid sequence of the purified protein was determined up to 10 amino acids as APQISPQYPM, exhibited high homology with that of alkaliphilic laccase from *M.verrucaria* 24G-4, APQISPQYPM [19] and bilirubin oxidase from *M. verrucaria* MT-1, VAQISPQYPM [20]. Peptides identified by MALDI-TOF of the protein revealed only 49% of homology with bilirubin oxidase from *M. verrucaria* (gi 2833236). These results showed the homospecificity of the protein with those from *M. verrucaria*, but not common laccases.

### Effects of temperature and pH value

**Figure 4:**
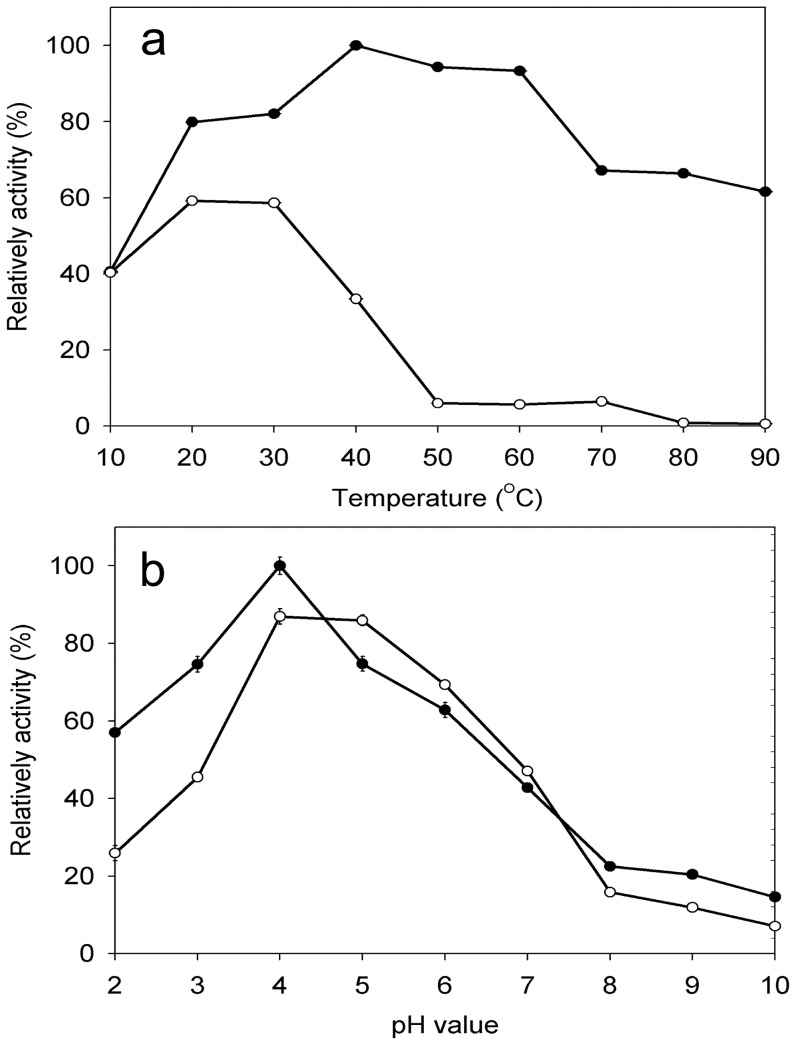
Optima and stability of (a) temperature and (b) pH value for purified laccase from *M.verrucaria* NF-05 reacting with ABTS. Optima curve (•); stability curve (○). The highest value of activity for each analytical curve was considered as 100%, and error bars shown are standard deviations of triplicate samples.

The purified laccase was more active in the temperature range of 20–60°C (Fig. 4a), which corresponded to the optimal temperature range for fungal laccase activity (30–60°C) indicated in the literature. The enzyme retained approximately 60% activity after incubation at 20–30°C for 1 h. However, the enzyme activity sharply decreased when the temperature was increased to 40°C and almost no activity was detected at 80°C. The laccase could still oxidise ABTS when incubated at 90°C for 3 min. However, the incubation of the enzyme without the substrate under the same condition demonstrated that the enzyme did not display any activity. The results revealed that the presence of the substrate protects the enzyme from being inactivated at high temperatures. Similar observations have been obtained in laccase from *Fusarium solani* [21], in cellulose [22] and in xylanase [23]. The pH profile for the laccase activity with the substrate ABTS showed a peak of maximum activity at pH 4.0 (Fig. 4b). The enzyme remained active at pH values from 2.0 to 7.0, and its activity at 2.0 and 7.0 were 57.0% and 42.8% of that at pH 4.0, respectively. The enzyme was stable over a pH range from 3.0 to 7.0 for 1 h and maintained for more than 40% activity. The preference for the acidic to neutral pH range is similar to most fungal laccases [2], [21] but is different form the alkaliphilic laccase that is produced by *M.verrucaria* 24G-4 [13].

### Effects of different organic solvent

**Figure 5:**
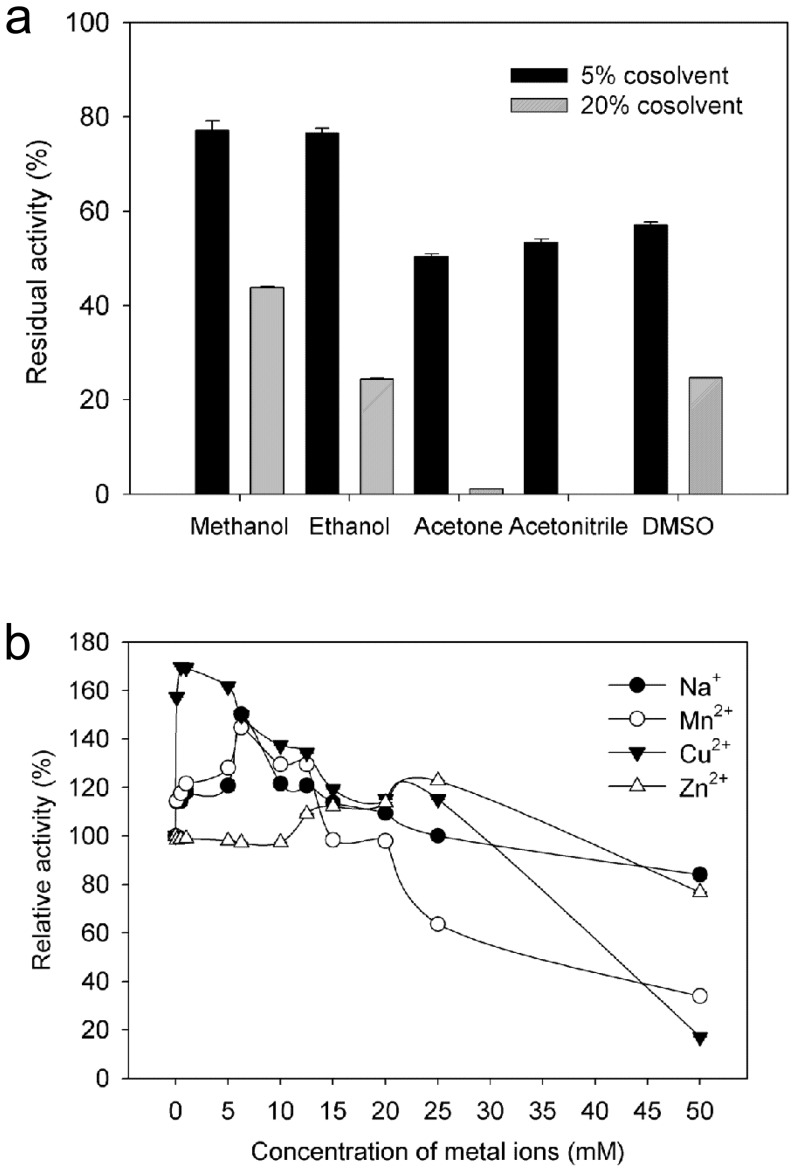
Effect of different organic solvents (a)and enhancement effects of metal ions (b) on the activity of purified laccase from *M.verrucaria* NF-05. All the experiments are performed with the same purified laccase, and activity without the addition of organic solvents was considered as 100%. Error bars shown are standard errors of triplicate samples.

The addition of water miscible organic solvents caused a decrease in the enzymatic activity by altering the pH of the aqueous solution. The purified laccase retained approximately 80% of its initial activity in the presence of 5% methanol and ethanol (Fig. 5a). These results indicated that the enzyme might be suitable for use in reactions that require a similar concentration of these solvents. However, the inhibitory effect increased with the increasing concentration of solvents. The activity was almost completely inhibited in the presence of 20% acetone and acetonitrile. The activity was completely inhibited when the concentration of all of the tested solvents increased to 50% (data not shown).

### Effects of metal ions and enzyme inhibitors

Metal ions especially heavy metal ions are common environmental pollutants and can affect the production and stability of the extracellular enzymes 1. Table 2 showed that the laccase activity was reduced or completely inhibited by Li^+^, K^+^, Ag^+^, Hg^+^, Mg^2+^, Ca^2+^, Cd^2+^, Fe^2+^, Co^2+^, Ba^2+^, Al^3+^, and Fe^3+^ at the tested concentrations of metal ions. The laccase activity was significantly increased by the presence of Na^+^ (6.25 mM, 150.2%), Zn^2+^, (25 mM, 122.75%) and Mn^2+^ (6.25 mM, 144.7%) (Fig. 5b). The laccase activity (0.5 mM, 169.7%) was enhanced by Cu^2+^ at a concentration of 0.1–25 mM. However, the laccase activity (17.2%) decreased when the Cu^2+^ concentration was raised to 50 mM (Fig. 5b). The large extent activation of activity by Cu^2+^ might be caused by the filling of type-2 copper binding sites with Cu^2+^ [13]. Many metal ions at specific concentrations have been reported to be activators for laccases such as Na^+^, Fe^2+^, Mn^2+^, Ba^2+^ [2], Mg^2+^, Zn^2+^, Fe^3+^ [24], Cu^2+^ [24], [25], Hg^2+^ [21] and Mo^6+^ [26].

**Table 2:** Effects of metal ions on laccase activity.

Metal ions	Residual activity (%)			
	6.25 mM	12.5 mM	25 mM	50 mM
Li^+^	41.9±0.2	19.8±0.2	17.8±0.2	6.4±0.1
K^+^	52.2±0.5	47.0±0.3	33.6±0.2	17.3±0.1
Ag^+^	8.3±0.6	8.1±0.1	8.6±0.1	6.9±0.1
Hg^+^	13.2±0.3	5.2±0.1	4.4±0.1	3.4±0.1
Mg^2+^	57.4±0.4	55.5±0.3	29.3±0.3	1.4±0.1
Ca^2+^	37.1±0.4	36.1±0.2	9.9±0.2	0
Cd^2+^	53.8±0.6	34.2±0.2	28.8±0.3	27.6±0.1
Fe^2+^	4.8±0.1	4.1±0.1	6.7±0.1	4.4±0.1
Co^2+^	49.1±0.2	28.3±0.2	4.4±0.1	0.4±0.1
Ba^2+^	13.5±0.3	14.2±0.2	9.5±0.1	0
Al^3+^	91.7±1.1	19.3±0.3	12.3±0.2	6.5±0.1
Fe^3+^	42.6±0.3	7.8±0.1	2.0±0.1	0

The enzymatic activity was completely inhibited by sodium azide (an inhibitor of oxidase), which suggested the function of laccase as an oxidase. DTT, which is a strong reducing agent on disulphide bonds, strongly inhibited the enzyme. These results indicate the existence of a disulphide structure in the active domain. L-cysteine and SDS caused complete inactivation of the enzyme. In addition, EDTA partially inhibited the laccase, which suggested that existence of a metal-binding domain in the protein. The enzyme revealed sensitivity to halogen anions, which are typical inhibitors of laccases (Table 3).

### Substrate and kinetic analysis

Table 4 showed the Michaelis-Menten constants for laccase in the presence of different substrates. The enzyme exhibited the highest activity with ABTS with the *K*m value of 85.9 µM, which was determined using the Lineweaver-Burk plot. The apparent *K*m values that were determined for hydroquinone, catechol and guaiacol were 2091.7, 329.3 and 312.2 µM, respectively. The *k*_cat_/*K*m value for ABTS was 3.1×10^6^ (s^−1^_·_M^−1^), which was higher than that for hydroquinone, catechol and guaiacol. These results indicated that ABTS had a more effective catalysis process. The laccase oxidised syringaldazine. However, the reaction rate was very low under the conditions that were provided. The affinity for ABTS [21], [24], [26] and the inaction to syringaldazine [21] have been observed in other fungal laccases.

### Degradation and colourisation of synthetic dyes

**Figure 6:**
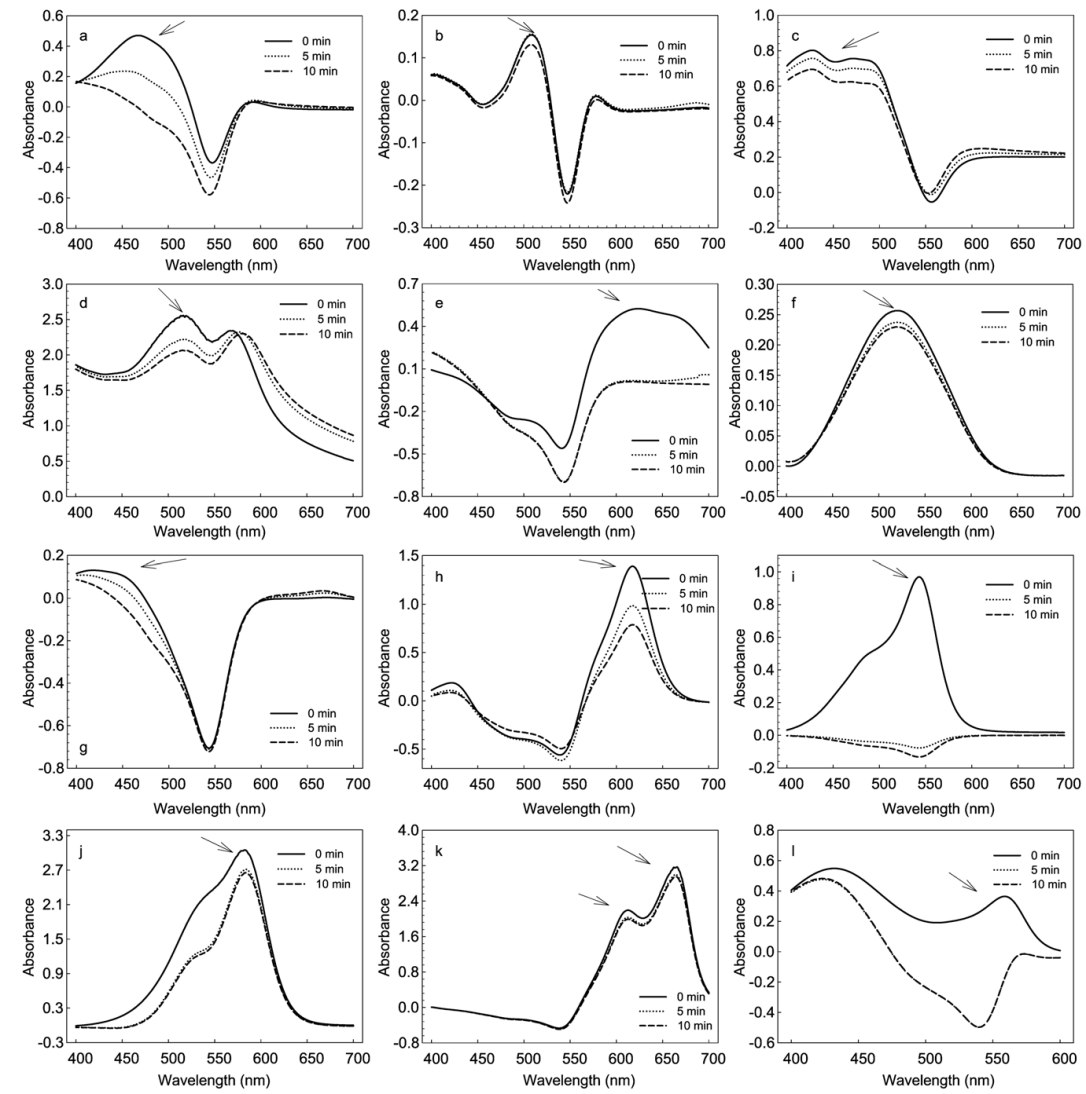
Degradation of azo dyes, a: orange I, b: amaranth, c: sudan II, d: sudan III, e: eriochrome black T; anthraquinone dyes, f: alizarin red, g: alizarin; arylmethane dyes, h: malachite green, i: fuchsin basic, j: crystal violet; and other dyes, k: methylen blue, l: phenol red by purified laccase from *M.verrucaria* NF-05.

Figure 6 showed the degradation of twelve structurally different dyes including azo, anthraquinone, arylmethyl and other structure type dyes by the purified laccase. Four out of twelve tested dyes, orange I, eriochrome black T, fuchsin basic and phenol red showed a total decolourisation by NF-05 laccase within 10 min. The remaining eight dyes were degraded to different extend within 24 h as revealed in table 5. There was no significant relation between structure and decolourisation efficiency of dyes. Though lots of papers reported the degradation and decolourisation of synthetic dyes by laccases [4], [10], the broad substrates specificity of NF-05 laccase rendered its great potentials in industrial applications, such as degradation of dyes from acidic textile effluents.

**Table 3:** Effect of inhibitors on laccase activity.

Inhibitor (mM)	Inhibition (%)	Inhibitor (mM)	Inhibition (%)
EDTA (0.1)	17.2±0.3	Cl^−1^ (20)	80.1±1.1
EDTA (1)	19.0±0.5	Cl^−1^ (200)	96.7±1.2
EDTA (5)	19.9±0.2	Cl^−1^ (800)	98.5±0.5
L-Cysteine (0.1)	83.1±0.7	Br^−1^ (20)	82.3±1.2
L-Cysteine (1)	100	Br^−1^ (200)	91.2±0.9
SDS (0.1)	56.1±0.3	Br^−1^ (800)	98.8±0.6
SDS (1)	77.8±0.3	I^−1^ (20)	88.4±0.8
SDS (5)	100	I^−1^ (200)	98.6±1.1
DTT (0.1)	96.8±0.3	I^−1^ (800)	99.1±1.2
DTT (1)	100		
NaN3 (0.1)	100		

**Table 4:** Substrate specificity of the purified laccase.

Substrate	*K*_m_ (µM)	*V*_max_ (µM min^−1^ mg^−1^)	*k*_cat_ (s^−1^)	*k*_cat_/*K*_m_ (s^−1^ M^−1^)
ABTS	85.9	555.6	267.1	3.1×10^6^
Hydorquinone	2091.7	3333.3	1587.3	7.6×10^5^
Catechol	329.3	1666.7	973.7	3.0×10^6^
Guciacol	312.2	416.7	198.4	6.4×10^5^

**Table 5:** Decolourisation of dyes by *M.verrucaria* NF-05 laccase.

Dyes	Decolourisation (%)		
	5 min	10 min	24 h
orange I	53.02%	100%	100%
Amaranth	0	14.72%	26.96%
sudan II	5.64%	13.36%	37.45%
sudan III	13.27%	19.29%	45.66%
eriochrome black T	96.78%	98.32%	99.45%
alizarin red	7.49%	10.51%	58.82%
Alizarin	19.84%	51.00%	80.26%
malachite green	29.32%	43.46%	56.33%
fuchsin basic	100%	100%	100%
crystal violet	11.02%	12.97%	36.25%
methylen blue	6.12%/7.46%	7.24%/9.25%	34.22%/45.36%
phenol red	100%	100%	100%

In conclusions, the purified ‘white’ laccase from the deuteromycete fungus *M.verrucaria* NF-05 was a monomeric protein displaying the typical properties as an oxidoreductase. The metal content and spectral properties indicated its novelty. The dye degradation ability added to the advantages that the organism and its enzyme possessed for bioremediation and biotransformation. Further studies should be focused on the analysis on the laccase-encoded gene, the mechanism of electron transfer in the active center of the laccase and also investigation on whether the laccase could be involved in the direct oxidization of different polycyclic aromatic hydrocarbons in vitro.

## Materials and Methods

### Microorganism and cultivation

Strain NF-05 was isolated from soil in Liangshui Native Nature Reserve, China and identified as *M.verrucaria* according to the internal transcribed spacer nucleotide sequence as well as the microscopic morphology based on the method of [18]. No specific permits were required for the described field studies. The fungus was monthly transferred to fresh PDA slants which contained (g l^−1^): potato, 200; glucose, 20; MgSO_4_·7H_2_O, 1.5; KH_2_PO_4_, 3; agar, 20 and stored at 30°C. For purification of the laccase, strain NF-05 was inoculated into the liquid PDA which contained (g l^−1^): potato, 200; glucose, 40; peptone, 35; MgSO_4_·7H_2_O, 1.5; KH_2_PO_4_, 3; pH 6.0. The culture medium was supplemented with 1 mM CuSO_4_·5H_2_O at the fourth day to induce the laccase production. Each flasks was then incubated with two fungal discs (1-cm diameter), which had been incubated 7–9 days earlier on PDA plates at 30°C. Each flasks (250 ml) contained 100 ml medium. Inoculated flasks were cultivated in a time course of 15 days.

### Protein and enzyme assays

The protein concentration was determined based on the Lowry procedure using bovine serum albumin (BSA) as the standard [27]. The activity of laccase was spectrophotometrically determined by applying ABTS (2,2′-azinobis-(3- ethylbenzothiazoline-6-sulphonic acid)) which was used as the substrate at 30°C in 0.2 M citric acid (pH 4.0). The reaction solution consisted of 2.95 ml of citric acid buffer (0.2 M, pH 4.0), 1 ml of ABTS (1 mM) and 50 µl of enzyme solution. The change in the absorbance due to the oxidation was monitored at 420 nm and recorded after 3 min. One unit (U) of activity was defined as the production of 1 µmol of product per minute under the condition of 30°C and pH 4.0. The activity of bilirubin oxidase was measured as follows: 2.0 ml of 30 µM bilirubin dissolved in 0.2 M Tris-H_2_SO_4_ buffer (pH 8.4) was added to 0.2 ml of the enzyme solution, followed by incubation at 37°C. Measurement of the absorbance decrease of bilirubin was carried out at 440 nm. One unit was defined as the amount of enzyme which oxidized 1 µM bilirubin min^−1^ [28]. The activity against hydroquinone, catechol, guaiacol and 4-hydroxy-3,5-dimethoxybenzaldehyde azine (syringaldazine) were also determined under the same reaction conditions with different initial concentrations. The absorbance coefficients were as follows: *ε*420 nm=36,000 M^−1^_·_cm^−1^ for ABTS, *ε*248 nm=10,400 M^−1^_·_cm^−1^ for hydroquinone, *ε*410 nm=2211 M^−1^_·_cm^−1^ for catechol, *ε*470 nm=6740 M^−1^_·_cm^−1^ for guaiacol, and *ε*525 nm=65,000 M^−1^ cm^−1^ for syringaldazine [16], [29]. One unit (U) of activity was defined as the production of 1 µmol product per minute under the condition of 30°C and pH 4.0.

### Enzyme purification

The laccase was purified from a cell-free culture medium by addition of ammonium sulfate to 70% saturation and centrifugation of salted-out proteins at 4°C, 10,000 g for 30 min. The supernatant was decanted and the precipitate was dissolved in 0.2 M citric acid buffer (pH 4.0) and dialyzed (membrane molecular weight cut off 14,000 Da) at 4°C overnight against the same buffer. The dialysate was loaded onto a DEAE-Cellulose column pre-equilibrated with the same buffer. Proteins were eluted with a linear gradient of sodium phosphate (0–0.8 M) in 10 mM citric acid buffer (pH 4.0) at a flow rate of 1 ml min^−1^. Fractions of 5 ml were collected, and those with laccase activity were pooled and concentrated using polyethylene glycol. The concentrate was then loaded onto a Sephadex G-75 column pre-equilibrated with the same buffer. Proteins were eluted with the same buffer at a flow rate of 0.5 ml min^−1^. Fractions with laccase activity were also pooled and the purified enzyme was stored at −20°C. The purification was carried out in the room temperature.

### Determination of molecular mass and metal content of purified laccase

The purity and molecular mass of the purified laccase was determined by SDS-PAGE using a 12% resolving gel and a 4% stacking gel. Proteins were visualised by staining with coomassie brilliant blue R-250. The unstained protein molecular weight marker was purchased from Fermentas (#SM0431) (Ontario, Canada). The zymogram process was performed by a native PAGE using a 12% resolving gel. The band of interest was visualised by incubating the gel in 0.2 M citric acid buffer containing 5 mM ABTS (pH 4.0) at 30°C. The metal content was determined by inductively coupled plasma atomic emission spectrometry (ICP-AES) using an Optima 7300DV, PerkinElmer.

### The spectrometric studies

The spectroscopic characterization of the purified enzyme was carried out in 10 mM citric acid buffer (pH 4.0) from 200 to 700 nm using a lambda750, PerkinElmer.

### Electron paramagnetic resonance (EPR)

An electron paramagnetic resonance (JEOL, Japan) spectrum was recorded with a JES-FA200 EPR spectrometer at 9.5 GHz, a modulation frequency of 100 kHz, a modulation amplitude of 2 G (200 µT), a sweep time/scan of 120 s and a microwave power of 5.0 mW. The enzyme sample was prepared in 10 mM citric acid buffer, pH 4.0. Probe temperature was regulated with a liquid nitrogen cryostat equipped with a temperature control unit and maintained at 100 K.

### N-terminal amino acid sequencing

The purified laccase was electrophoresed on 12% (w/v) SDS–PAGE, transferred onto a polyvinylidene fluoride membrane (Millipore Corp., Bedford, Mass.) by electroblotting and then stained. The polyvinylidene fluoride membrane slice containing the laccase was excised and sequenced on an ABI PROCISETM494cLC sequencer that employs Edman degradation to sequentially cleave and identify amino acids starting at the amino terminus (N-terminus) of the protein.

### Matrix assisted laser desorption/ionization-time of flight (MALDI-TOF)

After SDS-PAGE, the protein band corresponding to the zone of the enzyme was excised and washed three times with dd·H_2_O. The sample was decolored using 30% acetonitrile and 10 mM NH_4_HCO_3_, and the sample was then dehydrated with 100% acetonitrile and subsequently digested with 15 µl trypsin at 37°C for 16 h. The peptides were then extracted with 60% acetonitrile and 0.1% trifluoroacetic acid. 1 µl solution was used for further analysis. The sample was analyzed in reflectron mode using a 4800 Plus MALDI TOF/TOF™ Analyzer (Applied Biosystems, USA). Spectra were deisotoped using a detection threshold that was manually adjusted to exclude spectral noise, and the resulting peak list was used to search the NCBInr database using the GPS Explorer™ v 3.6.

### Enzyme characterization

The optimal temperature and the thermal stability were investigated using 0.2 M citric acid buffer (pH 4.0) with 1 mM ABTS as the substrate. The optimal temperature was determined from 10–90°C. The thermal stability was measured after incubating the proteins under different temperatures for 1 h before adding 1 mM ABTS-containing reaction buffer. The optimal pH value and the pH stability was determined in 0.2 M phosphate buffer (pH 2.0), 0.2 M citric acid buffer (pH 3.0–8.0), and 0.1 M glycine-NaOH buffer (pH 9.0–10.0). The stability at pH 2.0–10.0 was tested after incubating the enzyme for 1 h at 30°C. Metal ions (Li^+^, Na^+^, K^+^, Ag^+^, Hg^+^, Mg^2+^, Ca^2+^, Cd^2+^, Mn^2+^, Fe^2+^, Co^2+^, Zn^2+^, Ba^2+^, Cu^2+^, Al^3+^, Fe^3+^, each at 6.25, 12.5, 25 and 50 mM), inhibitors (EDTA, SDS, dithiothreitol (DTT), sodium azide, cysteine, 0.1, 1 and 5 mM; Cl^−1^, Br^−1^ and I^−1^, each at 20, 200 and 800 mM) and different organic solvents (methanol, ethanol, acetone, acetonitrile, DMSO, each at 5%, 20% and 50%) were mixed with the 1 mM ABTS-containing reaction buffer to obtain the respective final concentrations. The effect of these additives on the laccase activity was also determined by incubating the additives at 30°C for 1 h.

### Decolourisation of synthetic dyes

All the tested dyes were purchased from Sigma Company, detailed information was shown in Table 6. The degradation of twelve structurally different dyes by the purified laccase was determined by full spectrum scan among 400–700 nm at 5 min and 10 min, respectively. The decolorization of test dyes was calculated at 5 min, 10 min and 24 h, respectively. The reaction mixture for the standard assay contained respective dye (0.6 mg) in 10 mM citric acid buffer at pH 4.0 and the enzyme solution (15 U) in a total volume of 3 ml. The decolorization rate of dye, expressed as dye decolorization (%), was calculated as the formula: decolorization (%)=[(Ai−At)/Ai] * 100, where Ai: initial absorbance of the dye, At: absorbance of the dye along the time. All experiments were performed in triplicate.

**Table 6:** Characteristics of dyes tested in this work.

Dyes	Type	Chemical formular	λ_max_ (nm)
orange I	azo	C_16_H_11_N_2_NaO_4_S	467
Amaranth	azo	C_20_H_11_N_2_Na_3_O_10_S_3_	508
sudan II	azo	C_18_H_16_N_2_O	427
sudan III	azo	C_22_H_16_N_4_O	516
eriochrome black T	azo	C_20_H_12_N_3_NaO_7_S	624
alizarin red	anthraquinone	C_14_H_7_NaO_7_S·H_2_O	520
Alizarin	anthraquinone	C_14_H_8_O_4_	417
Malachite green	arylmethane	C_23_H_25_ClN_2_	617
fuchsin basic	arylmethane	C_20_H_20_ClN_3_	543
crystal violet	arylmethane	C_25_H_30_ClN_3_·9H_2_O	583
methylen blue	other	C_16_H_18_ClN_3_S·3H_2_O	666/613
phenol red	other	C_19_H_14_O_5_S	558
